# Meningomyelocele Perioperative Management in Neonatal: Case Series

**DOI:** 10.3390/children11101219

**Published:** 2024-10-07

**Authors:** Filla Reviyani Suryaningrat, Sindy Irenewati, Mirna Sobana, Fiva Aprilia Kadi, Aris Primadi, Tetty Yuniati

**Affiliations:** 1 Department of Child Health, Faculty of Medicine, Universitas Padjajaran, Dr. Hasan Sadikin General Hospital, Bandung 40161, Indonesia; 2 Faculty of Medicine, Universitas Diponegoro, Semarang 50244, Indonesia; 3 Department of Neurosugery, Faculty of Medicine, Universitas Padjajaran, Dr. Hasan Sadikin General Hospital, Bandung 40161, Indonesia

**Keywords:** myelomeningocele, neural tube defect, ruptured myelomeningocele, perioperative management, post operative complication

## Abstract

Background: Myelomeningocele (MMC) is a congenital malformation affecting the central nervous system, categorized as a neural tube defect (NTDs). In untreated cases, the mortality rate within the first six months of life ranges from 35% to 70%. Globally, its incidence is estimated 0.8 to 1 per 1000 live births, and rates are higher in Asia and lower socioeconomic groups. This disease imposes a significant treatment cost and burden due to life-long disabilities. In less developed or developing countries, delays in diagnosis can lead to complications such as infection and rupture. Although rupture is a rare complication in MMC, there are limited studies that have reported it. This study aims to discuss the perioperative management and potential complications of ruptured MMC. Method: This study presents four cases of ruptured MMC that were referred from private hospitals and managed by Hasan Sadikin General Hospital in West Java, Indonesia. Each patiens underwent a clinical assessment and diagnostic evaluation upon arrival, followed by perioperative theraphy and management of any complications that emerged during treatment. Result: In three of the four cases, the children were over 24 h old when they were admitted to the hospital. Three cases were located in the lumbosacral region while one case was located in the thoracic region. Upon arrival, the clinical presentations we observed included microcephaly, small for gestational age (SGA) and congenital talipes equionavrus (CTEV). And we found several complications included wound dehisence, respiratiory failure, hydrocephalus, leg weakness, menigitis and sepsis after surgery. Conclusions: Perioperative management is highlighted as vital, necessitating a multidisciplinary approach and precise surgical techniques to mitigate severe complications.

## 1. Introduction

Myelomeningocele is characterized by a midline cystic protrusion of the meninges and spinal cord through defective vertebral arches and covered by skin or a thin membrane [1]. This condition can manifest anywhere along the spine and is recognized as a severe form of spina bifida cystica, being the most common central nervous system (CNS) birth defect that is compatible with life. Without proper treatment, mortality rates within the first six months can range between 35% and 70% [2]. In terms of etiology, both genetic and environmental factors have been identified. Scientific data has demonstrated that intake of folic acid during the preconception phase is effective in reducing the risk of NTDs [3].

The global incidence of MMC ranges between 0.8 and 1 per 1000 live births. The incidence is several times higher in Asia and with lower socioeconomic status [2]. The disease places a significant financial and psychological impact on families due to the long-term disabilities it causes, such as paraplegia, incontinence, hydrocephalus, cardiovascular defects, and cognitive impairments [2,4].

In many developed countries, genetic counselling and folic acid supplementation during the pre-conception phase, together with accurate prenatal screenings, have significantly reduced the incidence of MMC [5]. However, in underdeveloped/developing countries, where adequate prenatal care is limited, the incidence remains higher and complications are more frequent. Although rupture is infrequent in MMC, very few studies have reported about the clinical presentation and the complications. This study presents 4 cases of ruptured MMC treated at Hasan Sadikin General Hospital in West Java, Indonesia and discusses the preoperative management and potential complications that may arise postoperatively.

## 2. Clinical Cases

### 2.1. Patient 1

A 25-h-old neonate, born at 35 weeks of gestation, to a mother gravida 4, para 4. There was no reported family history of NTDs or other congenital anomalies. The patient’s mother was doing routine antenatal care (ANC) controlled by the midwife at a primary health care, Bandung. The ANC provided includes the monitoring of body weight, blood pressure, upper arm circumference, fundal height, leopold maneuvers, fetal heart rate, blood tests (routine blood count, random blood glucose, triple elimination), and the administration of iron and folic acid supplements to be taken at home. Folic acid supplementation was initiated at an approximately 13 weeks of gestation. However, she was referred to a private hospital for a caesarean section due to suspected placenta previa, as there was a history of antepartum hemorrhage. This referral was not related to the presence of an MMC since the patient had never undergone an ultrasound (USG) examination and was therefore unaware that the fetus had been diagnosed with MMC.

**Figure 1:**
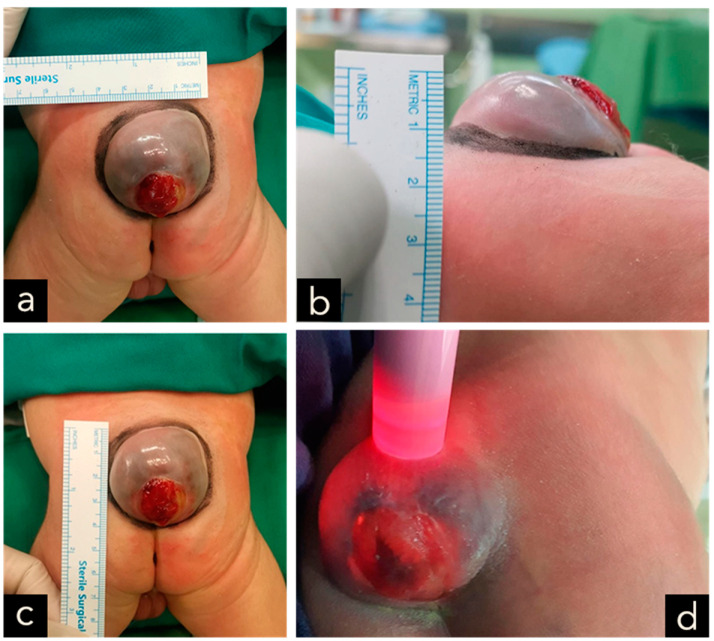
(**a**–**c**) A cystic MMC at the lumbosacral region, with skin erosion at the surface of the lesion, measuring 4 × 3 × 2 cm in size and associated with cerebrospinal fluid leakage. (**d**) Positive transillumination result.

**Figure 2:**
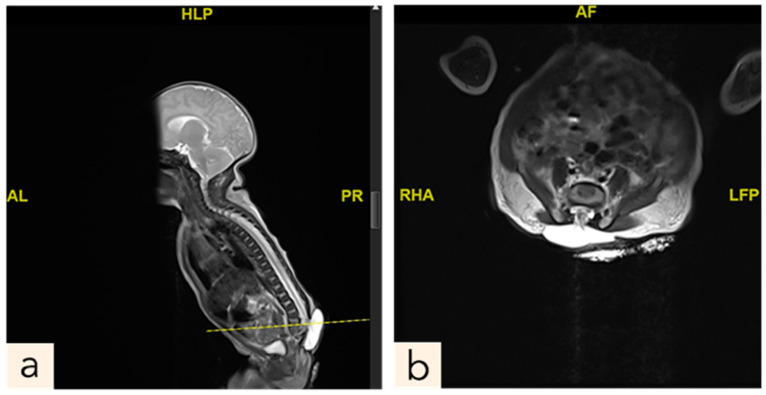
Contrast MRI pre-surgery, a Posterior lumbosacral mass at the level of L5-S1. (**a**) sagittal plane. (**b**) axial plane. (AL: for Anterior Left, HLP: Head of Lumbosacral Plexus, PR: Posterior Right, RHA: Right Hepatic Angle or Right Half of Abdomen, LFP: Left Fornix Posterior or Left Footplate, AF: Anterior Fontanelle).

The male neonate was born crying with a birth weight of 2685 g. The significant examination finding was a wound on the bulging of the lower back that does not enlarge when crying. The bulging size is 4 cm × 3 cm × 2 cm with minimal discharge and serous actively draining and positive transillumination result (Figure 1). The patient was diagnosed with suspected ruptured meningomyelocele by neurosurgery in Private Hospital and then referred to the Neurosurgery Emergency Room at Hasan Sadikin General Hospital Bandung. Upon admission to our hospital, the patient had already voided and defecated. There was no evidence of macrocephaly or any clinical signs suggestive of hydrocephalus. The symptoms were not accompanied by seizures, decreased consciousness, fever, no weakness in the limbs was observed. Magnetic Resonance Imaging (MRI) done at 3 days old diagnosing a meningomyelocele at regio lumbosacral described as a cystic mass accompanied by a component of the spinal cord within it at a level of vertebra corpus L5-S1 (Figure 2).

Before undergoing surgery, the neonate received comprehensive pre-operative care, which included the following measures: blood laboratory test (results are shown in Table 1), swab sampling of discharge for culture examination and administration of fluids and nutrients at a rate of 60–150 mL/kgbw per day, gradually increased over five days, consisting of breast milk or infant formula. Additionally, the antibiotic ampicillin and gentamicin was administered for a period of five days. The patient’s condition was stable, with no signs of sepsis, seizures, or limb weakness. At 7 days old (6 days of hospital care) there was a combined surgery to do a myelomeningocele repair by the Neurosurgery and Plastic Surgery teams.

Postoperatively, the patient was managed in a prone position with the head lowered and the pelvis elevated in an inpatient setting. Fluids and nutrition were administered at a rate of 120–150 mL/kgbw per day, consisting of parenteral nutrition combined with breast milk or infant formula, gradually increasing over a 28-day hospital stay. The patient received antibiotic meropenem for 7 days, following the results of a pus culture, that identified Staphylococcus haemolyticus which was resistant to a range of antibiotics, including ampicillin and gentamicin. The antibiotic regimen continued by administration of ampicillin-sulbactam for 14 days due the results of the lumbar cerebrospinal fluid (CSF) culture from a specimen obtained during surgery, that identified Acinetobacter baumanii which was resistant to a range of antibiotics including gentamicin and meropenem. Blood tests were conducted routinely to monitor for metabolic issues (results are shown in Table 1). Daily head circumference measurements and regular monitoring of signs of increased ICP were performed, along with head ultrasound examinations as needed to monitor the development of hydrocephalus.

**Figure 3:**
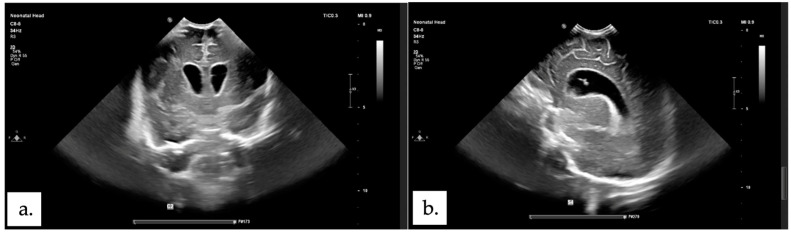
Post-surgical head ultrasound showing ventriculomegaly. (**a**) axial plane. (**b**) sagittal plane.

**Figure 4:**
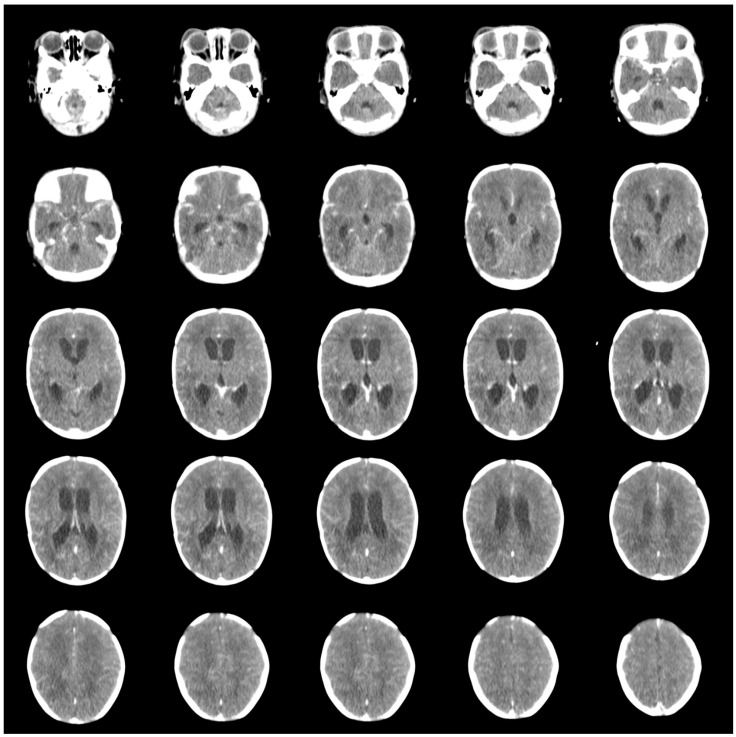
Post-surgical head CT scan showing enlargement of all ventricle system and periventricular hypodensity.

The patient encountered multiple complications following the surgical procedure. On the first postoperative day, a seizure was due to electrolyte imbalance (Na 127 and K 7.2) occurred and by the fifth postoperative day, blood tests revealed a drop in hb (haemoglobin) level to 10.6 and hypoalbuminemia (2.7). An ultrasound examination was performed on the sixth postoperative day to evaluate the condition, revealing ventriculomegaly (Figure 3). On the seventh postoperative day, a CT scan was conducted, identifying hydrocephalus. Despite the absence of a rapid increase in head circumference (Pre-op: 33 cm, POD-7: 33.2 cm) and no signs of decreased consciousness, the CT scan revealed periventricular hypodensity (Figure 4). The Neurosurgery team managed this by inserting an EVD. The patient experienced seizures on the second day of EVD placement, along with turbid yellow EVD output prompting an USG examination that indicated ventriculitis. Treatment included the addition of antibiotics amikacin and metronidazole for 14 days. Respiratory failure was diagnosed on the tenth postoperative day, requiring transfer to the Neonatal Intensive Care Unit (NICU) for six days for monitoring and ventilatory support. Thirteen days postoperatively, while in the NICU, the patient developed sepsis, and the antibiotic regime was continued until 14 days, followed by administration of levofloxacin and vancomycin (intraventricular) for the remaining 7 days due the results of the lumbar CSF culture, which identified the presence of Klebsiella pneumoniae. On the sixteenth postoperative day, the patient was transferred out of the NICU, requiring continued oxygen therapy via nasal cannula for five days to address intermittent seizures. The EVD was removed after the CSF culture results showed no bacterial growth and there were no signs of increased intracranial pressure. The patient was released home at 35 days old and 28 days post-operative and the surgical scar remained clean without discharge. The parents were educated on how to monitor their child’s overall condition and measure head circumference daily at home. The patient was planned to have a follow-up at the Neurosurgery polyclinic, Neonatology polyclinic and the Pediatric Infections Diseases polyclinic at Hasan Sadikin General Hospital Bandung.

### 2.2. Patient 2

A 3-h-old neonate, born at 39 weeks of gestation, to a mother gravida 5, para 5. There was no reported family history of NTDs or other congenital anomalies. The patient’s mother only underwent one ANC controlled by a midwife at primary health care. The ANC provided includes the measurement of body weight, blood pressure, upper arm circumference, fundal height, leopold maneuvers and fetal heart rate. However, she was referred to a private hospital for a caesarean section due to breech position, asthma and hypertension. This referral was not related to the presence of an MMC as the patient had never undergone an ultrasound (USG) examination and had no history of folic acid supplementation, and was therefore unaware that the fetus had been diagnosed with MMC.

**Figure 5:**
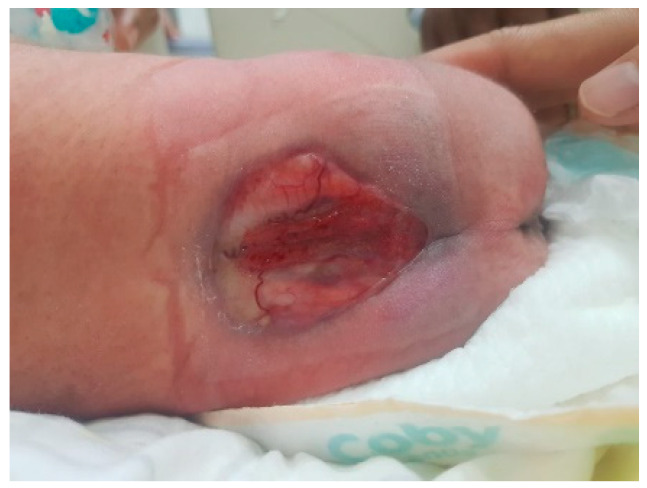
MMC with an open wound at the lumbosacral region, with skin erosion, measuring 4 × 7 × 0.5 cm in size and associated with cerebrospinal fluid leakage.

The male neonate was born crying with a birth weight of 2420 g, categorized as low birth weight and small for gestation. The significant examination finding was a microcephaly with a head circumference of 30 cm and an open wound at the lower back. The lesion size is 4 cm × 7 cm × 0.5 cm with minimal discharge and serous actively draining found in the lumbosacral region (Figure 5). The patient was diagnosed with suspected ruptured meningomyelocele by a paediatrician in Private Hospital and then referred to the Neurosurgery Emergency Room at Hasan Sadikin General Hospital Bandung. Upon admission to our hospital, the patient had already voided and defecated. The symptoms were not accompanied by seizures, decreased consciousness, fever and no weakness in the limbs was observed.

Before the surgical procedure, the neonate underwent thorough pre-operative preparation, which included the following measures: blood laboratory test (results are shown in Table 2), swab sampling of discharge for culture examination and administration of fluids and nutrients at a rate of 30–60 mL/kgbw per day, consisting of breast milk or infant formula. Furthermore, the patient was administered the antibiotic ampicillin and gentamicin from the start of hospitalization until the surgical repair. The patient’s condition remained stable, showing no signs of sepsis, seizures, or limb weakness. At 2 days old (1 day of hospital care), a combined myelomeningocele repair surgery was performed by the Neurosurgery and Plastic Surgery teams.

Postoperatively, the patient was managed in a prone position with the head lowered and the pelvis elevated in an inpatient setting. Fluids and nutrition were administered at a rate of 60–150 mL/kgbw per day, consisting of parenteral nutrition combined with breast milk or infant formula, gradually increasing over a 34-day hospital stay. Antibiotics ampicillin and gentamicin were continued for 7 days. Blood tests were conducted routinely to monitor for metabolic issues (results are shown in Table 2). Daily head circumference measurements and regular monitoring of signs of increased ICP were performed, along with head ultrasound examinations as needed to monitor the development of hydrocephalus.

**Figure 6:**
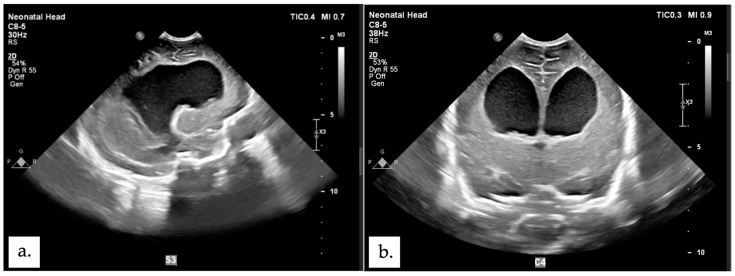
Post-surgical head ultrasound showing ventriculomegaly. (**a**) sagittal plane. (**b**) axial plane.

**Figure 7:**
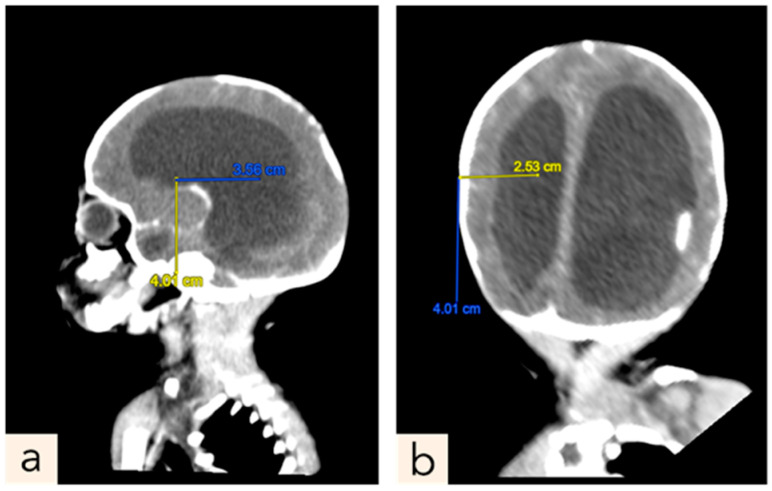
Contrast Head CT scan post-surgery, enlargement of all ventricle system. (**a**) sagittal plane. (**b**) axial plane.

The patient encountered multiple complications following the surgical procedure. On the eight days postoperative, bilateral leg weakness was observed, persisting until discharge. Concurrently, periorbital edema and hypoalbuminemia (2.2) were identified through clinical examination and blood tests, respectively. After 7 days of treatment with ampicillin and gentamicin, the antibiotic regimen was continued with cefotaxime and amikacin for 14 days, followed by ceftazidime for the remaining 13 days. On the nineteenth postoperative day, a TORCH screening was conducted, resulting in a diagnosis of Rubella. By the twenty-first postoperative day, a decrease in hemoglobin levels was noted (9.3), suspected to be attributable to an infectious process. During this period, the patient also presented with wound dehiscence. On the same day, the patient experienced respiratory distress which improved with the administration of oxygen via binasal canula. Therefore an orogastric tube (OGT) was placed to assist with feeding. There were no signs of increased intracranial pressure after surgery and the lumbar CSF culture obtained during surgery showed no microbial growth. The patient was released home at 33 days old and 31 days post-operative closure defect—with orogastric tube (OGT) due to feeding problem, and the surgical scar remained clean with minimal wound dehiscence. The parents were educated on how to monitor their child’s overall condition and measure head circumference daily at home. Fifteen days later, the patient was readmitted at 48 days old (POD 46) due to experiencing six episodes of seizures at home and the mother reporting a rapid increase in the child’s head circumference (birth: 30 cm, POD 1: 30.7 cm, POD 31: 33.1, POD 46: 37.1). An USG (Figure 6) and CT scan (Figure 7) was performed, revealing obstructive hydrocephalus. At 53 days old (POD 51), the patient underwent a Ventriculoperitoneal shunt (VP shunt) procedure. The patient later planned to have a follow-up at the Neurosurgery polyclinic, Neonatology polyclinic and the Pediatric Infections Diseases polyclinic at Hasan Sadikin General Hospital Bandung.

### 2.3. Patient 3

A 31-h-old neonate, born at 39 weeks of gestation, to a mother gravida 1, para 1. There was no reported family history of NTDs or other congenital anomalies. The patient’s mother was not doing routine ANC. She was referred to a private hospital for a caesarean section due to complete breech. This referral was not related to the presence of an MMC since the patient had never undergone an ultrasound (USG) examination and had no history of folic acid supplementation, and was therefore unaware that the fetus had been diagnosed with MMC.

**Figure 8:**
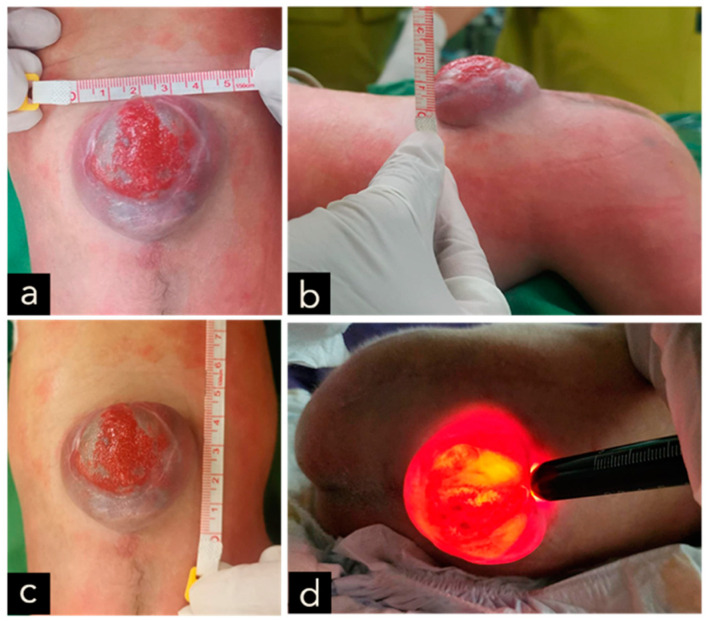
(**a**–**c**) A cystic MMC at the lumbosacral region, with skin erosion at the surface of the lesion, measuring 4 × 3 × 1 cm in size and associated with cerebrospinal fluid leakage. (**d**) Positive transillumination result.

**Figure 9:**
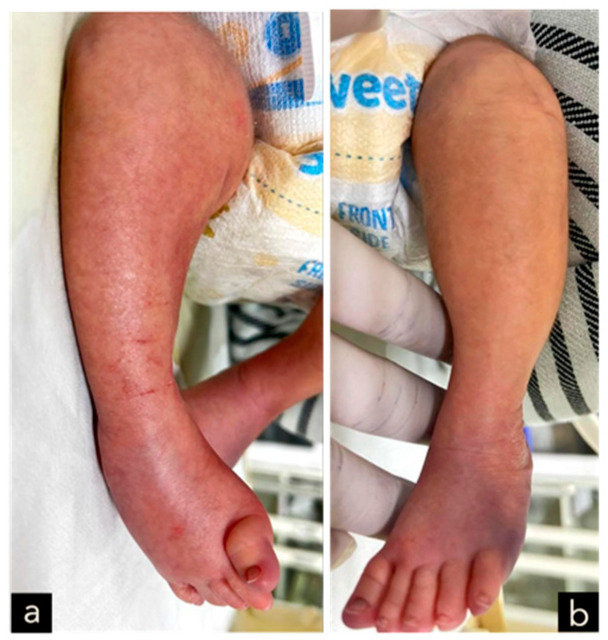
A deformity of CTEV at the right leg. (**a**) right leg. (**b**) left leg.

The male neonate was born crying with a birth weight of 3100 g. The significant examination finding was a wound on the bulging of the lower back that enlarged when crying. The bulging size is 4 cm × 3 cm × 1 cm with minimal discharge and serous actively draining and positive transillumination result found in the lumbosacral region (Figure 8). The patient also had a deformity of CTEV on his right leg (Figure 9). The patient was diagnosed with suspected ruptured meningomyelocele by a paediatrician in Private Hospital and then referred to the Neurosurgery Emergency Room at Hasan Sadikin General Hospital Bandung. Upon admission to our hospital, the patient had already voided and defecated. The symptoms were not accompanied by seizures, decreased consciousness, and fever. There was no evidence of macrocephaly or any clinical signs suggestive of hydrocephalus.

Before undergoing surgery, the neonate received comprehensive pre-operative care, which included the following measures: blood laboratory test (results are shown in Table 3), swab sampling of discharge for culture examination and administration of fluids and nutrients at a rate of 60–150 mL/kgbw per day, consisting of breast milk or infant formula. Additionally, the patient received the antibiotic ampicillin and gentamicin from the beginning of hospitalization until the time of surgical repair. The patient’s condition remained stable, with no evidence of sepsis, seizures, or limb weakness. At 9 days old (6 days care of hospital care) there was a combined surgery to do a meningomyelocele repair by the Neurosurgery and Plastic Surgery teams.

Postoperatively, the patient was managed in a prone position with the head lowered and the pelvis elevated in an inpatient setting. Fluids and nutrition were administered at a rate of 120–150 mL/kgbw per day, consisting of parenteral nutrition combined with breast milk or infant formula, gradually increasing over a 19-day hospital stay. The antibiotic treatment was continued with ceftazidime and amikacin for 7 days, following the results of a pus culture, which identified the presence of Enterobacter cloacae. The culture sensitivity testing revealed that the bacterium was sensitive to amikacin. Conversely, it is resistant to a range of antibiotics, including ampicillin, and gentamicin. This was then followed by ampicillin sulbactam and vancomycin for the final 13 days, as the patient was diagnosed with bacterial meningitis on the seventh postoperative day as indicated by lumbar CSF culture from a specimen obtained during surgery that showed a complex infection profile. Lumbar CSF cultures identified Acinetobacter baumannii and Escherichia coli which showed resistance to a wide range of antibiotics including ampicillin, gentamicin, amikacin and ceftazidim. Furthermore, the patient was diagnosed with sepsis around the same time frame as the blood culture findings revealed the presence of Staphylococcus equorum, which was resistant to antibiotics such as penicillin G, ampicillin, and gentamicin. Blood tests were conducted routinely to monitor for metabolic issues (results are shown in Table 3). Daily head circumference measurements and regular monitoring of signs of increased ICP were performed, along with head ultrasound examinations as needed to monitor the development of hydrocephalus.

On the thirteenth postoperative day, impairment in the patient’s legs was observed, leading to a diagnosis of paraplegia affecting the lower extremities. The patient did not require respiratory support during the hospital stay and there were no signs of increased intracranial pressure. The patient was discharged home at 26 days old and 19 days post-operative closure defect and the surgical scar remained clean without discharge. The parents were educated on how to monitor their child’s overall condition and measure head circumference daily at home. The patient later planned to have a follow-up at the Neurosurgery polyclinic, Neonatology polyclinic and the Pediatric Infections Diseases polyclinic, Pediatric neurology polyclinic to follow up the neurological impairment and orthopaedic polyclinic to treat the CTEV at Hasan Sadikin General Hospital Bandung.

### 2.4. Patient 4

A 36-h-old neonate, born at 39 weeks, to a mother gravida 1, para 1. There was no reported family history of NTDs or other congenital anomalies. The patient’s mother was doing routine ANC controlled by a midwife at primary health care. The ANC provided includes the monitoring of body weight, blood pressure, upper arm circumference, fundal height, leopold maneuvers, fetal heart rate, blood tests (routine blood count, random blood glucose, triple elimination), and the administration of iron and folic acid supplements to be taken at home. Folic acid supplementation was initiated at an approximately 14 weeks of gestation. However, she was referred to a private hospital for a caesarean section due to suspected oligohydramnions. This referral was not related to the presence of an MMC since the patient had never undergone an ultrasound (USG) examination and was therefore unaware that the fetus was diagnosed with MMC.

**Figure 10:**
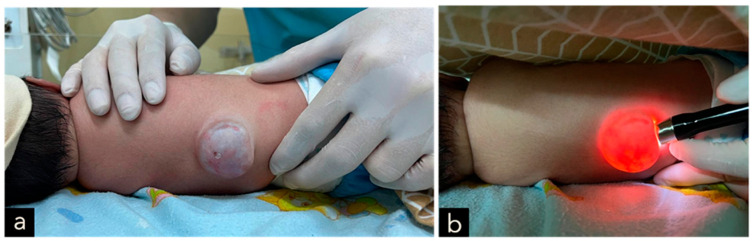
(**a**) A cystic MMC at the thoracic region, with a hole accompanied by CSF leakage on the upper part of the lesion, measuring 3.5 × 3.5 × 1.5 cm in size and associated with cerebrospinal fluid leakage. (**b**) Positive transillumination result.

**Figure 11:**
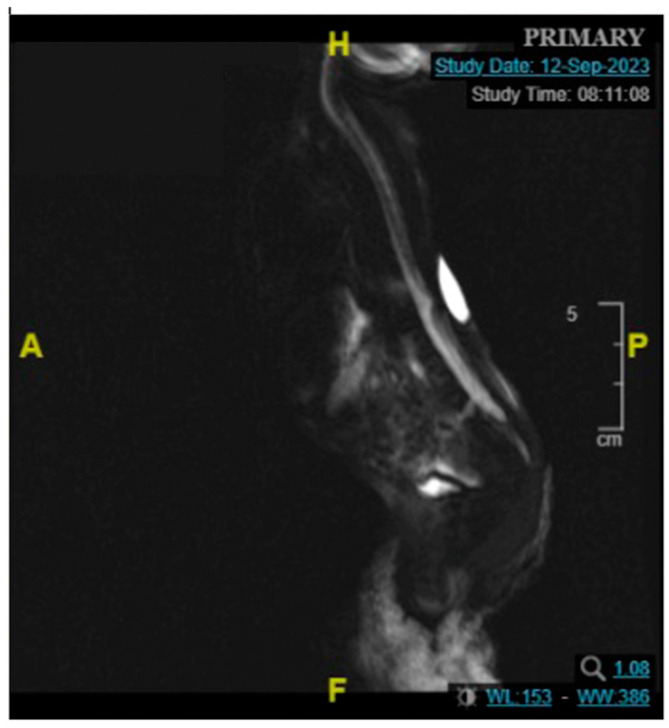
Contrast MRI pre-surgery sagittal plane, a posterior thoracic mass at the level of T12.

The male neonate was born crying with a birth weight of 2400 g, categorized as low birth weight. The significant examination finding was a wound on the bulging of the lower back that does not enlarge when crying. The bulging size is 3.5 cm × 3.5 cm × 1.5 cm with minimal discharge and serous actively draining (Figure 10). The patient was diagnosed with suspected ruptured meningomyelocele by a paediatrician in Private Hospital and then referred to the Neurosurgery Emergency Room at Hasan Sadikin General Hospital Bandung. Upon admission to our hospital, the patient had already voided and defecated. There was no evidence of macrocephaly or any clinical signs suggestive of hydrocephalus. The symptoms were not accompanied by seizures, decreased consciousness, fever and no weakness in the limbs was observed. MRI done at 3 days old diagnosing a meningomyelocele at thoracic region described as a cystic mass accompanied by a component of the spinal cord within it at a level of vertebra corpus T12 (Figure 11).

Before undergoing surgery, the neonate received comprehensive pre-operative care, which included the following measures: blood laboratory test (results are shown in Table 4), swab sampling of discharge for culture examination and administration of fluids and nutrients at a rate of 60–120 mL/kgbw per day, consisting of breast milk or infant formula and the patient was managed in a prone or side-lying position. Additionally, the antibiotic ampicillin and gentamicin was administered for a period of two days. The patient’s condition was stable, with no signs of sepsis, seizures, or limb weakness. At 4 days old (3 days of hospital care) there was a combined surgery to do a meningomyelocele repair by the Neurosurgery and Plastic Surgery teams.

Postoperatively, the patient was managed in a prone position with the head lowered and the pelvis elevated in an inpatient setting. Fluids and nutrition were administered at a rate of 120–160 mL/kgbw per day, consisting of parenteral nutrition combined with breast milk or infant formula, gradually increasing over a 28-day hospital stay. Fluids and nutrition were administered at a rate of 120–150 mL/kgbw per day, consisting of parenteral nutrition combined with breast milk or infant formula over a post-operative 4-day hospital stay. The antibiotic treatment Ampicillin and gentamicin was continued for 4 days. The patient did not experience sepsis, meningitis, or limb weakness after the surgery. Blood tests were conducted routinely to monitor for metabolic issues (results are shown in Table 4). Daily head circumference measurements and regular monitoring of signs of increased ICP were performed, along with head ultrasound examinations as needed to monitor the development of hydrocephalus.

The patient did not experience complication such as sepsis, meningitis, or limb weakness after the surgery. The patient did not require respiratory support during the hospital stay, there were no signs of increased intracranial pressure and the lumbar CSF culture obtained during surgery showed no microbial growth. The patient was discharged home at 8 days old and 4 days post-operative closure defect and the surgical scar remained clean without discharge. The parents were educated on how to monitor their child’s overall condition and measure head circumference daily at home. The patient was planned to have a follow-up at the Neurosurgery polyclinic and Neonatology polyclinic at Hasan Sadikin General Hospital Bandung.

## 3. Discussion

Myelomeningocele is a congenital malformation affecting the central nervous system characterized by the herniation of the meninges and spinal cord containing cerebrospinal fluid through a bony defect in the skull, categorized as NTDs [5]. Myelomeningocele represents the most prevalent form of spina bifida, accounting for 86.8% of cases, as reported in one particular study [1]. The incidence of MMC is estimated at 0.8 to 1 per 10,000 live births; though this rate differs based on geographic region and race [5].

The underlying causes of myelomeningocele remain unclear to this day. However, the risk factors for MMC have been identified, such as genetics, prior neural tube defect, dietary and socioeconomic status. Approximately 5% of MMC cases are found in families with a known family history, whereas 95% arise spontaneously in children of women without a familial history [1]. Similar to the four cases we have presented, these four babies do not have a family history of NTDs. Women who have previously given birth to a child with MMC have a 3–8% higher risk of having another child with the same condition.

Maternal folic acid deficiency has been linked to a two-to-eight-fold increase in the risk of having a child with MMC [2]. In the cases we present, two out of four mothers only initiated ANC and began taking folic acid at 13/14 weeks of pregnancy. This suggests that they likely did not undergo health examinations during pregnancy planning and only started folic acid supplementation after ANC commenced upon confirmation of pregnancy. Meanwhile, the remaining two neither engaged in routine ANC nor consumed folic acid throughout her pregnancy. This could be attributed to a combination of inadequate health seeking behaviours and low socioeconomic status, considering that Indonesia is a developing country with diverse socioeconomic statuses.

The Centers for Disease Control and Prevention (CDC) recommends supplementation of 400 mcg of folic acid starting one month before pregnancy and 4000 mcg starting three months before pregnancy for women who have previously given birth to a child with MMC [6]. A cohort study from China reported that FA supplementation successfully reduces the prevalence of NTD, with a rate of 6.4 per 1000 births in mothers who did not consume folic acid, compared to 1.3 per 1000 births in those who did [7]. Another study reported folic acid deficiency in mothers of children with MMC, leading to the finding that pre-conception folic acid supplementation could significantly reduce the incidence of myelomeningoceles by up to 70% [1,8]. In planned pregnancies, routine antenatal folic acid supplementation reduces the likelihood of deficiency, unlike in unplanned pregnancies where supplementation is less common. Therefore, the risk of having a child with MMC is significantly lower in planned pregnancies [1]. According to World Health Organization (WHO) data, there are 200 million pregnancies annually in Indonesia, with 75 million, or 38%, being unintended [9]. Consequently, this suggests a likelihood of low folic acid consumption among pregnant women in Indonesia.

Myelomeningocele can occur in different types of anatomical location. A study involving 352 cases reported the following locations: cervical (1.8%); cervicothoracic (0.9%); thoracic (4.2%); thoracolumbar (17.4%); lumbar (16.8%); lumbosacral (22.3%); sacral (34.5%); and rachischisis present in 7 (2.1%) of subjects [1]. Three out of four cases we presented feature MMC located in the lumbosacral area, which is the second most common location for MMC occurrences based on a recent study. Interestingly, a study showed that MMC located in the thoracic or thoracolumbar region was associated with a higher infection (*p* = 0.007; *p* = 0.036), while underlying mechanism remain unclear [10].

The clinical presentation of spina bifida varies based on its type and severity. Myelomeningocele typically presents as a midline defect in the lumbosacral region, where the skin is deficient. Upon initial examination, the region typically looks ulcerated and moist, with a visible placode containing primitive neuronal epithelium. A case example from our study is provided in Figure 1, Figure 5, Figure 7 and Figure 9.

Myelomeningocele is a form of dysraphism which may lead to various degrees of morbidity, such as lower limb motor impairments, hydrocephalus, bladder and anal sphincter disturbances, orthopedic disorders, and sexual dysfunction [11]. Before the surgery, on our case, we found microcephaly, lower limb motor impairment and orthopaedic findings such as CTEV.

In our 2nd case, we identified a baby with clinical features of microcephaly and a diagnosis of rubella. Maternal infections play a key role in adverse obstetric outcomes. A study mentioned 200 TORCH (toxoplasmosis, other agents, rubella, cytomegalovirus, and herpes simplex virus) cases; where 24 cases found to have congenital anomalies, with rubella being the most common cause and MMC being one of the anomalies identified [12].

In our 3rd case, we identified a patient with congenital talipes equinovarus (CTEV), which is commonly associated with MMC. The deformity and neurological deficits in MMC vary according to lesion level with CTEV potentially occurring at any level; however, the most frequently reported lesion involves the mid-lumbar region at L3 and L4 [13]. This was similar to the third case where the MMC was located at the lumbosacral level. CTEV in MMC is considered to develops during the intrauterine period due to early foot agenesis, causing muscle contractions and fibrosis due to the disruption of typical intrauterine muscle movements. Patients with MMC may also present with clubfoot deformity due to prolonged neuromuscular deficits [13].

Screening NTDs can be performed through fetal ultrasound at 11 to 14 weeks of gestation, where MMC can be detected before the 12th postmenstrual week by spotting structural abnormalities in the spinal bones or a bulging along the posterior outline of the fetal back. The detection of open spina bifida is reported to be 80–90% [3]. In our study, all four mothers lacked USG examinations. This led to a lack of preparedness among these mothers in managing children with meningomyelocele cases. Inadequate management and delayed referral to larger hospitals can result in ruptured MMCs and subsequent infections.

Cases of ruptured MMC are rarely reported in the literature. Across numerous developed countries, preventive strategies including genetic counselling, periconceptional folic acid supplementation, prenatal screening and diagnosis, and the legalisation of therapeutic abortion have played a key role in reducing the prevalence of NTDs. As a result, ruptured and superinfection is extremely unusual. Meanwhile, underdeveloped countries and even in certain developed ones, where pregnancy monitoring and preventive measures are lacking and interventions are delayed, these conditions may still occur [5].

MRI is the preferred method for characterizing NTDs, as it plays a key role in diagnosis, identifying associated malformations, therapeutic planning and postoperative monitoring due to excellent spatial resolution and enhanced tissue contrast without the use of ionizing radiation. Postnatal MRI of untreated MMC may reveal nerve roots originating from the ventral surface of the placode, extending through the MMC and entering the dilated subarachnoid spaces of the spinal canal, reaching the neural foramina [14]. However, MRI in newborns with NTDs especially MMC is rarely performed, as the diagnosis is typically made obstetric ultrasound and confirmed visually after birth.

Treatment of MMC requires surgical resection, with the appropriate technique chosen based on the location of the malformation. Several factors must be considered before surgery, including the patient’s general condition, any associated anomalies, and the features of the MMC, including its size and location. The primary goal of treatment is to reintegrate the herniated contents and close the meningeal defect [5].

In our study, before the MMC repair, comprehensive pre-operative care was provided to all the neonates [15]. The neonates were positioned prone or side-lying and left unclothed, wearing only a loose nappy to facilitate proper care. Despite ruptured MMC in all cases, the lesions were covered with moistened, non-adherent gauze and a non-permeable dressing to prevent fluid loss, drying, and protect the CSF from contamination. In addition, steps were made to prevent stool from contaminating the lesion as much as possible. The neonates were cared for in a controlled thermal environment to keep their axillary temperature between 36.5 °C and 37.5 °C, ensuring their comfort and safety during this critical pre-operative period. The neonates were administered maintenance fluids which gradually increased during their hospital stay. Prophylactic broad-spectrum antibiotics were given from birth until surgical repair to reduce the risk of infection.

In our study, the neonates were administered maintenance fluids at a rate of 30–150 mL/kgbw/day, which gradually increased during their hospital stay. This approach aligns with the fluid requirements of neonates from the first day to beyond seven days. For full-term neonates weighing more than 1500 g, the fluid requirement on the first day is around 30 mL/kgbw/day, which can be progressively increased to 150 mL/kgbw/day as the neonate’s age exceeds one week [16].

In the last 10 years, prophylactic antibiotics were being used for all MMC patients throughout the whole observation period, it became a recommended procedure consisting of prophylactic ampicillin (150 mg/kg/d i.v. twice daily) and gentamicin (5 mg/kg/d i.v. once daily on day one, 4 mg/kg/d i.v. once daily starting from day 2) for 5 days beginning at the day of birth before the surgery. Post surgical infection following MMC closure is a common complication, with infection rates are up to 36% without prophylactic antibiotic treatment [17]. Similar in our cases, all neonates with MMC received ampicillin and gentamicin as antibiotics prophylaxis before the surgery.

Management of NTDs uniquely depends on each case. In open NTDs, the concern is to close the exposed neural elements and neural placode as soon as possible, as well as evaluate the CSF leakage, the movement of extremities, spinal reflex, and the existence of hydrocephalus. Reconstruction of the neural placode was done by detailed inspection and suturing up of the placode, arachnoid, and dura layers individually in watertight fashion, followed by fascia and skin [18]. In the presence of a CSF leak, surgical closure needs to be performed within the first 24 h. If no CSF leak detected, surgical closure can be safely extended for up to 48 h [19]. For closed NTDs, neuroimaging evaluation for tethered cord and soft tissue adhesion was needed for future surgical approaches [18]. Three out of four cases presented underwent surgery more than 48 h post-admission. This delay is attributed to the initial suspicion of contamination in the defect area, as the patients presented to the hospital at over 24 h of age, necessitating preliminary management prior to confirming the absence of infection. Proceeding with surgery in the presence of an infection raises concerns of severe complications.

Similarly, following the surgery, comprehensive post-operative care was provided to all neonates in our study [15]. This involved assessing airway support and closely monitoring temperature, as there is a risk of the neonate becoming hypothermic intraoperatively. The post-operative orders and instructions provided by the neurosurgeon in the operative notes were meticulously followed. For 7–10 days post-operation, neonates were nursed either prone or side-lying to avoid pressure on the suture lines and to enhance wound healing. The wound was covered with a transparent dressing, and care was taken to assess the wound regularly for any signs of leakage, wound dehiscence, or infection. When soiling was observed, the dressing was changed to prevent the risk of wound infection.

Postnatal MMC repair, however, is linked to various complications. A study mentions that complications happened in their cases, including wound infection (7%), despite the MMC repair performed during the early stage, CSF leakage from the surgical site (39%), pyogenic meningitis (19%), ventriculitis (4.9%), shunt infection (9.8%) and weakening of lower limb function (2.9%) [3]. Our study found several complications that happen post-surgical, such as wound dehiscence, sepsis, meningitis, and respiratory failure.

In our second case, the neonate experienced wound dehiscence post-surgery. Wound complications, including dehiscence and CSF leak, may be seen after neurosurgical correction of MMC. A study reported that 11% of cases experienced wound infection postoperatively [3]. Another study indicated that out of 156 neonates, 21 patients (13.5%) developed surgical site infection, while 135 patients (86.5%) showed satisfactory wound healing. Furthermore, this study revealed that 37 patients (23.7%) experienced CSF leakage, whereas 119 patients (76.3%) achieved complete recovery [20]. Factors influencing wound infection include several causes, one of which is the defect surface area (DSA). A study mentioned that 9.1% of wound infections occurred with a DSA less than 26 cm^2^, compared to a 50% infection rate in DSAs larger than 50 cm^2^ [3].

The current study highlighted post-operative hypoalbuminemia plays a critical role in increasing the risk of wound complications [20]. In our first and second cases, the neonates experienced hypoalbuminemia, where case one presented with sepsis and case two with wound dehiscence. Albumin deficiency is known to prolong the inflammatory phase of wound healing, reduce fibroblast numbers, inhibit proteoglycan and collagen synthesis, disrupt the neoangiogenesis process, and impair wound healing. A study has reported that post-surgical patients often experience hypoalbuminemia, with 56.4% having albumin levels under 3.5 g/dL and 20.6% under 3.0 g/dL within the first seven days after surgery. Furthermore, an albumin level under 3 g/dL after surgery was associated with a doubled risk (Odds Ratio [OR] = 2.003) of surgical site infection and a fivefold increase (OR = 5.219) in the risk of surgical wound dehiscence [21]. This was consistent with our findings, where both cases with hypoalbuminemia had albumin levels at 3.0 g/dL. However, several factors affect the reduction of postoperative albumin. A study noted that the surgical stress response, fluid overload during surgery, hemodilution, redistribution of albumin, metabolic breakdown, and other associated factors cause postoperative albumin decline [22].

Hyponatremia is one of the most frequent electrolyte imbalances observed in the perioperative phase, with a serious potential complication being hyponatremic encephalopathy, which may lead to irreversible neurological damage or death. The likelihood of experiencing symptoms, including seizures and abnormal neurological findings, increases when sodium levels drop below 125 mEq/L. Post-operative patients are prone to electrolyte derangements, primarily due to factors such as blood and fluid loss, the body’s stress response to surgery, the administration of intravenous fluids, blood transfusions, and the underlying surgical disease [23,24]. Interestingly, the rate of hyponatremia is remarkably more common in neonates, with studies from neonatal intensive care units revealing an average incidence rate of 40% [25]. Preventative measures, such as fluid restriction and the use of isotonic fluids, have been shown to mitigate the risk of hyponatremia in ill children [24]. Additionally, research indicates that neonates with hyponatremia are significantly more likely to develop various outcomes such as sepsis, as we found in our 1st case.

In our first case, ventriculitis occurred one day after the insertion of an EVD. Cerebrospinal fluid infections including ventriculitis associated with neurosurgery and intracranial devices like EVDs, are common and serious complications that significantly extend hospital length of stay and drive up healthcare costs [3]. A recent study from a French neurosurgical intensive care unit (ICU) performing routine daily swabs of skin at EVD insertion site, EVD stopcock, and CSF cultures, revealed that ventriculitis largely arises from “extra-luminal progression of pathogens initially colonizing the skin site where CSF leaks” [17]. CSF leaks were highlighted as a major risk factor for ventriculitis [26].

In our first and third cases, the neonates developed sepsis. Sepsis is a known complication in some cases, as evidenced by a study conducted over three years, which recorded 60 consecutive patients with myelomeningocele. Out of total 60 patients, 2 (3.3%) patients developed sepsis after undergoing neurosurgical intervention [2]. Most authors agree that surgical closure performed more than 48 h post-delivery poses a significant risk for wound sepsis and other related complications [3,27].

A study involving 53 neonates who underwent MMC repair surgery within 72 h of life found that 18.2% developed sepsis, a higher rate than previous studies reported [28]. Another research on 314 children treated for MMC revealed that 58 out of 314 (21%) developed post-surgical sepsis, with an average onset of wound sepsis occurring at 9.5 ± 3.6 days. This study also identified wound sepsis as key factor impacting both of mortality rates and length of hospital stay. The average length of hospital stay for children with wound sepsis was 38.1 ± 22.3 days, while those without sepsis had a stay of 20.4 ± 16.9 (*p* = 0.002) [29].

Furthermore, post-neurosurgical patients with sepsis require closer monitoring, as sepsis has been identified as an independent predictor of mortality up to one-year post-discharge. While sepsis is relatively rare in this context, neurosurgical patients developing sepsis after elective surgery have notably worse outcomes than those who do not. These outcomes involve longer hospital stays, increased readmission rates, and higher risk of mortality [30].

In our 1st case, the neonates required NICU care for respiratory failure, that was compounded or may have been induced by the presence of sepsis. The mechanism linking respiratory failure to sepsis involves several critical factors, including enhanced permeability of pulmonary capillaries, alveolar epithelial cells dysfunction, and inflammatory cells infiltration. The diffusing alveolar injury, marked by endothelial cell dysfunction and localised inflammation, stands as the pathological signature of this condition. This dysfunction in the microvascular endothelium of the alveoli impairs oxygen transport and exchange, resulting in severe, treatment-resistant hypoxia. With sepsis, the patient’s immune system becomes dysregulated, accelerating pro-inflammatory signalling that exacerbates vascular endothelial dysfunction and promotes additional inflammatory cells. This intense systemic inflammation primarily results in the hyperpermeability of the pulmonary microvasculature due to sepsis, causing the alveoli to fill with plasma exudate, thereby exacerbating respiratory failure [29].

In case 2 of our study, the patient exhibited weakness in both legs at eight days postoperatively, persisting until hospital discharge. It’s crucial to assess neurological function in children post-MMC repair, as neurological dysfunction is likely as they grow older [31]. A study has highlighted that children who have undergone MMC repair may experience tethered cord syndrome (TCS), a clinical syndrome characterized by progressive neurological, orthopaedic, and urological symptoms. This condition is often caused by an abnormal spinal cord attachment to the surrounding scar tissue or operative scar [32]. Furthermore, a study observing 42 children post-operative MMC repair over 18 months reported that 52.4% of patients were ambulatory but spastic, 21.4% were non-ambulatory, and 4.8% were bedridden [31].

Hydrocephalus is frequently associated with open NTDs and up to 80% of patients with MMC and Chiari Il malformation require cerebrospinal fluid diversion for managing the hydrocephalus [32]. Hydrocephalus associated with MMC can occur both preoperatively and postoperatively. In the preoperative phase, it may be caused by several genes controlling PCP (planar cell polarity) that have been shown to be associated with both neural tube defects and hydrocephalus [33]. However, in our study, we observed that in case 1 and case 2, hydrocephalus developed postoperatively. A study reported that 35 patients (22.4%) developed post-operative hydrocephalus, while 121 patients (77.6%) did not experience hydrocephalus postoperatively [20]. This might happen due to failure in sufficient CSF absorption after the primary MMC sac repair [34]. The treatment of CSF diversion in hydrocephalus associated with NTDs has not changed over the years, including shunt placement and endoscopic third ventriculostomy [35]. The timing of shunt placement was still debatable, but some studies showed that combining shunt insertion with NTD repair in the same procedure was generally more beneficial for the patients [36,37]. Routine brain ultrasound and neuroimaging studies should be conducted along with careful examination of the fontanelle and head circumference to determine if immediate CSF diversion is necessary [32]. After surgery, it is essential to observe for signs of hydrocephalus [15]. In our cases, we performed daily head circumference measurements and monitored for signs of increased ICP, while conducting head ultrasounds and CT scans as needed. An EVD was placed if there was progressive increased in ICP or brain parenchyma/ventricle wall damage, as confirmed by CT scan in our cases. The EVD is evaluated within 3–5 days, and if there are no signs of increased ICP, no marked increase in head circumference, and the CSF culture is negative, the EVD can be removed. If the patient becomes EVD-dependent, a VP shunt is placed.

Perioperative management plays a crucial role in the treatment of MMC, requiring strong multidisciplinary collaboration. While early MMC repair and the implantation of a ventriculoperitoneal (VP) shunt can significantly reduce morbidity and mortality, several postoperative complications remain common. The most important causes of postoperative morbidity and mortality include CSF leakage, VP shunt infection and dysfunction, surgical wound infection, and extensive scarring at the surgical site. Therefore, continuous post-operative monitoring is essential to detect and manage potential complications. We outline the recommended perioperative management strategies to address these challenges effectively, as shown in Table 5.

The study limitation is the absence of prenatal data from the mothers, which is partly due to the significant number of pregnant women in Indonesia who do not receive adequate ANC. Additionally, there is insufficient long-term follow-up data on the patients, limiting the assessment of future neurological outcomes. Further studies are needed to enhance prenatal monitoring practices and provide comprehensive long-term follow-up for patients post-surgery.

## 4. Conclusions

Perioperative management is crucial in addressing MMC in neonates to prevent severe complications such as infection, respiratory failure, and motor dysfunction. A multidisciplinary approach and precise surgical techniques are necessary to optimize patient outcomes. Despite effective perioperative care, high rates of mortality, morbidity, and long-term neurological deficits remain significant concerns.

## Figures and Tables

**Table 1:** Blood laboratory tests result—Patient 1.

Examination	Pre-Operative	Post-Operative						Reference Range	Unit
		Day-1	Day-5	Day-8	Day-10	Day-18	Day-26		
Hemoglobin	14.1	12.7	10.6	15.7	14.3	13.8	13.4	13.5–21.5	g/dL
Hematocrit	39.4	34.3	30.3	44.1	41.4	46	38.5	42.0–66.00	%
Leukocytes	14.39	29.74	20.59	32.56	35.82	10.72	9.05	13.0–38.0	10^3^/uL
Platelets	324	528	680	594	646	400	255	150–450	10^3^/uL
C-Reactive Protein (CRP)	<0.1	0.1	1.73	1.84	2.67	0.3	0.1	<0.3	mg/dL
Random Blood Glucose	52	73	71	88	170	81	76	70–140	mg/dL
Sodium (Na)	136	127	133	133	133	131	131	135–145	mEq/L
Potassium (K)	5.2	7.2	5.9	4.6	4.5	4.7	4.6	4.5–5.6	mEq/L
Albumin	3.05	2.9	2.7	3.2	3.2	3.0	3.0	3.8–5.4	g/dL
SGOT (AST)	28							15–37	U/L
SGPT (ALT)	8							0–55	U/L
Ureum	10.1							10.9–35.9	mg/dL
Creatinine	0.39							0.72–1.25	mg/dL
Lactic acid					2.6			1.1–3.7	mmol/L
pH					7.37			7.32–7.49	
pCO_2_					34.9			26.0–41.0	mmHg
pO_2_					72.1			60.0–70.0	mmHg
HCO_3_					20.5			16.0–24.0	mmol/L
tCO_2_					21.6			23.05–27.35	mmol/L
BE					−3.4			(−2)–(+3)	mmol/L
SpO2					93.9			95–100	%

**Table 2:** Blood laboratory tests result—Patient 2.

Examination	Pre-Operative	Post-Operative					Reference Range	Unit
		Day-1	Day-8	Day-14	Day-21	Day-29		
Hemoglobin	16.8	14	11.5	11.5	9.3	11.9	13.5–21.5	g/dL
Hematocrit	48.9	40.7	33.5	33.9	27.6	35.8	42.0–66.00	%
Leukocytes	15,360	8660	12,340	23,950	14,940	15,790	13.0–38.0	10^3^/uL
Platelets	144,000	238,000	396,000	381,000	492,000	575,000	150–450	10^3^/uL
C-Reactive Protein (CRP)	<0.1	0.11	0.47	1.07	1.11	0.18	<0.3	mg/dL
Random Blood Glucose	72	75	82	93	81	79	70–140	mg/dL
Sodium (Na)	134	135	140	135	133	134	135–145	mEq/L
Potassium (K)	5.5	5.2	4.9	5.0	5.2	4.8	4.5–5.6	mEq/L
Albumin	3.4	3.2	2.22	3.54	2.8	3.1	3.8–5.4	g/dL
SGOT (AST)	31						15–37	U/L
SGPT (ALT)	19						0–55	U/L
Ureum	16.2						10.9–35.9	mg/dL
Creatinine	0.78						0.72–1.25	mg/dL

**Table 3:** Blood laboratory tests result—Patient 3.

Examination	Pre-Operative	Post-Operative				Reference Range	Unit
		Day-1	Day-4	Day-9	Day-19		
Hemoglobin	17.6	16.4	13.5	11.7	12.8	13.5–21.5	g/dL
Hematocrit	49.9	47.4	40.1	32.7	36	42.0–66.00	%
Leukocytes	14,970	26,670	26,630	28,400	17,130	13.0–38.0	10^3^/uL
Platelets	302,000	628,000	609,000	614,000	625,000	150–450	10^3^/uL
C-Reactive Protein (CRP)	0.46	0.92	9.4	2.72	0.4	<0.3	mg/dL
Random Blood Glucose	92	109	133	62	81	70–140	mg/dL
Sodium (Na)	138	136	140	134	133	135–145	mEq/L
Potassium (K)	5	4.6	4.9	5.2	5.2	4.5–5.6	mEq/L
Albumin	3.17	3.91	3.14	3.13	3.1	3.8–5.4	g/dL
SGOT (AST)	30					15–37	U/L
SGPT (ALT)	9					0–55	U/L
Ureum	15.9					10.9–35.9	mg/dL
Creatinine	0.57					0.72–1.25	mg/dL

**Table 4:** Blood laboratory tests result—Patient 4.

Examination	Pre-Operative	Post-Operative		Reference Range	Unit
		Day-1	Day-8		
Hemoglobin	14.2	14.4	13.0	13.5–21.5	g/dL
Hematocrit	40.4	41	37.1	42.0–66.00	%
Leukocytes	8850	11,670	10,430	13.0–38.0	10^3^/uL
Platelets	325,000	313,000	450,000	150–450	10^3^/uL
C-Reactive Protein (CRP)	<0.1	<0.1	<0.1	<0.3	mg/dL
Random Blood Glucose	92	78	81	70–140	mg/dL
Sodium (Na)	139	132	133	135–145	mEq/L
Potassium (K)	5.3	6.1	5.1	4.5–5.6	mEq/L
Albumin	3.3	3.1	3.1	3.8–5.4	g/dL
SGOT (AST)	37			15–37	U/L
SGPT (ALT)	8			0–55	U/L
Ureum	12.3			10.9–35.9	mg/dL
Creatinine	0.5			0.72–1.25	mg/dL

**Table 5:** Recommendation.

Pre-Operative Management: Positioning Stabilization of the lesion Blood laboratory tests Swab sampling of discharge for culture examination Administration of fluids and nutrients Prophylactic administration of broad-spectrum antibiotics Diagnostic evaluation with MRI
Operative Management: Surgical repair of the spinal lesion for neonates with myelomeningocele should be performed within the first 24–72 h of life. Intra-operative CSF sampling for culture
Post-Operative Management: Wound care Cardiorespiratory monitoring Close monitoring of body temperature Routine blood tests for the monitoring and management of metabolic issues Administration of Fluids and nutrition: Parenteral nutrition should be administered until the neonate is clinically stable, after which enteral feeds should be commenced and gradually increased. Administration of therapeutic antibiotics according to culture results Regular observation of head circumference, signs of increased intracranial pressure, and cranial ultrasound as needed to monitor for hydrocephalus Monitoring for potential complications Family education: Ensuring that parents and families are adequately supported is a critical priority in both the pre- and post-operative care of neonates with myelomeningocele.

## Data Availability

Data are contained within the article.
